# Livestock Biosecurity from a One Health Perspective

**DOI:** 10.3390/ani14223309

**Published:** 2024-11-18

**Authors:** Claude Saegerman, Véronique Renault

**Affiliations:** 1 Research Unit of Epidemiology and Risk Analysis Applied to Veterinary Sciences (UREAR-ULiège), Fundamental and Applied Research for Animal and Health (FARAH) Center, Department of Infections and Parasitic Diseases, Faculty of Veterinary Medicine, University of Liege, 4000 Liège, Belgium; 2 VSF International, One Health, LEARN Project, 1210 Brussels, Belgium; v.renault@vsf-international.org

## 1. Introduction

According to the World Health Organization and the Food and Agriculture Organization of the United Nations, biosecurity is “a strategic and integrated approach that encompasses the policy and regulatory frameworks (including instruments and activities) that analyse and manage risks in the sectors of food safety, public health, animal life and health, and plant life and health, including associated environmental risk”. Biosecurity is a key component of any animal and public health strategy, as well as of disease prevention and control programs.

This Special Issue on livestock biosecurity from a One Health perspective focuses especially on practical aspects at the farm and community level, as well as at different country income levels (low-, middle-, and high-income countries).

## 2. The Structure of This Special Issue

Special Issue includes five papers illustrating the new challenge of livestock biosecurity from a One Health perspective. The first paper, written by Renault et al. [1], introduces the biosecurity concept by reviewing its origins and evolution and discusses the future perspectives of biosecurity in regard to the One Health Approach and the changing environment. Under the COST Action “Biosecurity Enhanced Through Training Evaluation and Raising Awareness” (BETTER) CA20103, the second paper, written by Saegerman et al. [2], evaluates the extent to which this framework agrees with nine existing definitions on biosecurity, with a focus on livestock. The by far most popular biosecurity definition was the one that conceptualized the rule of the 5Bs (bio-exclusion, bio-containment, bio-compartmentation, bio-prevention, and bio-preservation). The survey results highlight the need for the integration of more aspects into the existing definitions of livestock biosecurity (especially the prevention of zoonoses and preservation of the environment and diversity). The third paper, written by Militzer et al. [3], highlights the need for initiatives such as the FAO Progressive Management Pathway for Terrestrial Animal Biosecurity (FAO-PMP-TAB), which is a stepwise approach for strengthening the biosecurity management along value chains to enhance the health, resilience, and sustainability of animal sectors. The fourth paper, written by Pedersen et al. [4], uses existing knowledge to develop a novel approach to comprehensively assess the overall (mainly external) on-farm biosecurity level by a trained biosecurity assessor. This approach can be used for systematic data collection in epidemiological studies on risk factors for the introduction and establishment of *S.* Dublin in dairy farms. The fifth paper, written by Viltrop et al. [5], describes and analyzes the difference between biosecurity levels and management practices of ASF outbreaks (less favorable) and uninfected herds (more favorable) in order to identify potential risk factors for ASF introduction. Reducing the number of contacts between the farm and the external environment should be emphasized in a situation where ASF is circulating in wild boar populations close to pig farms.

## 3. Conclusions

This Special Issue highlights a variety of innovative contributions (from history and conceptualization to practical use) to provide information on practical mitigation options for better One Health livestock biosecurity. Further work is needed on the production of innovative and affordable tools to assess the level of biosecurity and engage farmers and vets in the fight against livestock biosecurity threats. Further attention should be devoted to low- and middle-income countries, and several initiatives should be promoted, such as pilot studies in accordance with the FAO Progressive Management Pathway for Terrestrial Animal Biosecurity.
